# Rényi Entropy and Rényi Divergence in Product MV-Algebras

**DOI:** 10.3390/e20080587

**Published:** 2018-08-08

**Authors:** Dagmar Markechová, Beloslav Riečan

**Affiliations:** 1 Department of Mathematics, Faculty of Natural Sciences, Constantine the Philosopher University in Nitra, A. Hlinku 1, SK-949 01 Nitra, Slovakia; 2 Department of Mathematics, Faculty of Natural Sciences, Matej Bel University, Tajovského 40, SK-974 01 Banská Bystrica, Slovakia; 3 Mathematical Institute, Slovak Academy of Sciences, Štefánikova 49, SK-814 73 Bratislava, Slovakia

**Keywords:** product MV-algebra, partition, Rényi entropy, conditional Rényi entropy, Rényi divergence

## Abstract

This article deals with new concepts in a product MV-algebra, namely, with the concepts of Rényi entropy and Rényi divergence. We define the Rényi entropy of order *q* of a partition in a product MV-algebra and its conditional version and we study their properties. It is shown that the proposed concepts are consistent, in the case of the limit of *q* going to 1, with the Shannon entropy of partitions in a product MV-algebra defined and studied by Petrovičová (*Soft Comput.* **2000**, *4*, 41–44). Moreover, we introduce and study the notion of Rényi divergence in a product MV-algebra. It is proven that the Kullback–Leibler divergence of states on a given product MV-algebra introduced by Markechová and Riečan in (*Entropy* **2017**, *19*, 267) can be obtained as the limit of their Rényi divergence. In addition, the relationship between the Rényi entropy and the Rényi divergence as well as the relationship between the Rényi divergence and Kullback–Leibler divergence in a product MV-algebra are examined.

## 1. Introduction

The Shannon entropy [1] and Kullback–Leibler divergence [2] are the most significant and most widely used quantities in information theory [3]. Due to their successful use, many attempts have been done to generalize them. It is known that their important generalizations are the Rényi entropy and Rényi divergence [4], respectively. These quantities have many significant applications; for example, in statistics, in ecology, and also in quantum information.

Shannon’s entropy is defined in the context of a probabilistic model in the following way: if we consider a probability space (Ω,S,P) and a measurable partition ℰ $=\left\{E_{1},E_{2},\ldots ,E_{n}\right\}$ of (Ω,S,P), then the Shannon entropy of ℰ is defined as the number H(ℰ)$=-\sum_{i=1}^{n}P(E_{i})\cdot \log P(E_{i})$ (with the usual convention that $P(E_{i})\cdot \log P(E_{i})=0,$ for $P(E_{i})=0).$ If ℰ$=\left\{E_{1},E_{2},\ldots ,E_{n}\right\}$ and ℱ$=\left\{F_{1},F_{2},\ldots ,F_{m}\right\}$ are two measurable partitions of (Ω,S,P), then the conditional Shannon entropy of ℰ assuming a realization of ℱ is defined as the number H(ℰ/ℱ) $=-\sum_{i=1}^{n}\sum_{j=1}^{m}P(E_{i}\cap F_{j})\cdot \log\frac{P(E_{i}\cap F_{j})}{P(F_{j})}$ (with the usual convention that $0\cdot \log\frac{0}{x}=0$ if x≥0)*.* If ℰ $=\left\{E_{1},E_{2},\ldots ,E_{n}\right\}$ is a measurable partition of (Ω,S,P) with probabilities $p_{i}=P(E_{i}),$ i=1,2,…,n, then its Rényi entropy of order q, where q∈(0,1)∪(1,∞), is defined as the number $H_{q}\left(ℰ\right)$ $=\frac{1}{1-q}\log\sum_{i=1}^{n}p_{i}^{q}.$ It can be shown that $\underset{q\to 1}{\lim}H_{q}$ (ℰ) $=\sum_{i=1}^{n}p_{i}\cdot \log\frac{1}{p_{i}},$ thus the Shannon entropy is a limiting case of the Rényi entropy for q→1. It is known that there is no universally accepted definition of conditional Rényi entropy. The paper [5] describes three definitions of conditional Rényi entropy that can be found in the literature. In [6], it is also possible to find a brief overview of various approaches to defining the conditional Rényi entropy, and in addition, a new definition of conditional Rényi entropy was proposed. In [7], the authors introduced a general type of conditional Rényi entropy which contains some of previously defined conditional Rényi entropies as special cases. The proposed concepts have successfully been used in information theory [8], time series analysis [9], and cryptographic applications [10]. However, no one of the proposed generalizations satisfies all basic properties of Shannon conditional entropy. The selection of the definition therefore depends on the purpose of application.

The present article is devoted to the study of Rényi entropy and Rényi divergence in product MV-algebras. An MV-algebra [11] is the most useful instrument for describing multivalued processes, especially after its Mundici’s characterization as an interval in a lattice ordered group (cf. [12,13]). At present, this algebraic structure is being studied by many researchers and it is natural that there are results also regarding entropy in this structure; we refer, for instance, to [14,15]. Also, a measure theory (cf. [16]) and a probability theory (cf. [17]) were studied on MV-algebras. Of course, in some problems of probability it is necessary to introduce a product on an MV-algebra, an operation outside the corresponding group addition. The operation of a product on an MV-algebra was suggested independently in [18] from the viewpoint of mathematical logic and in [19] from the viewpoint of probability. Also, the approach from the viewpoint of a general algebra suggested in [20] seems interesting. We note that the notion of a product MV-algebra generalizes some classes of fuzzy sets; a full tribe of fuzzy sets (see e.g., [21]) presents an example of a product MV-algebra.

A suitable entropy theory of Shannon and Kolmogorov-Sinai type for the case of a product MV-algebra has been provided by Petrovičová in [22,23]. We remark that in our article [24], based on the results of Petrovičová, we proposed the notions of Kullback–Leibler divergence and mutual information of partitions in a product MV-algebra. In the present article, we continue studying entropy and divergence in a product MV-algebra, by defining and studying the Rényi entropy and the Rényi divergence.

The rest of the paper is structured as follows. In the following section, preliminaries and related works are given. Our main results are discussed in Section 3, Section 4 and Section 5. In Section 3, we define the Rényi entropy of a partition in a product MV-algebra and examine its properties. It is shown that for q→1 the Rényi entropy of order *q* converges to the Shannon entropy of a partition in a product MV-algebra introduced in [22]. In Section 4, we introduce the concept of conditional Rényi entropy of partitions in a product MV-algebra and study its properties. It is shown that the proposed definition of the conditional Rényi entropy is consistent, in the case of the limit of *q* going to 1, with the conditional Shannon entropy of partitions studied in [22], and that it satisfies the property of monotonicity and a weak chain rule. In the final part of this section, we define the Rényi information about a partition *X* in a partition *Y* as an example for the further usage of the proposed concept of the conditional Rényi entropy. Section 5 is devoted to the study of Rényi divergence in a product MV-algebra. It is shown that the Kullback–Leibler divergence in a product MV-algebra introduced by the authors in [24] can be obtained as the limit of the Rényi divergence. We illustrate the results with numerical examples. The last section contains a brief summary.

## 2. Preliminaries and Related Works

We start by reminding the definitions of basic terms and some of the known results that will be used in the article. We mention some works related to the subject of this article, of course, without claiming completeness.

Several different (but equivalent) axiom systems have been used to define the term of MV-algebra (cf., e.g., [19,25,26]). In this paper, we use the definition of MV-algebra given by Riečan in [27], which is based on the Mundici representation theorem. Based on Mundici’s theorem [12] (see also [13]), MV-algebras can be considered as intervals of an abelian lattice-ordered group (shortly *l*-group). We remind that by an *l*-group (cf. [28]) we understand a triplet (G,+,≤), where (G,+) is an abelian group, (G,≤) is a partially ordered set being a lattice and x≤y⇒x+z≤y+z.

**Definition** **1** ([27])**.** *An MV-algebra is an algebraic structure* A =(A,⊕,∗,0,u) *satisfying the following conditions:*

*(i)*  *there exists an l-group*(G,+,≤)*such that*A=[0,u]={x∈G;0≤x≤u},*where 0 is the neutral element of*(G,+)*and u is a strong unit of G (i.e.,*u∈G*such that*u>0*and to every*x∈G*there exists a positive integer*n*with the property*x≤nu;
*(ii)*  ⊕*and*∗*are binary operations on A satisfying the following identities:*x⊕y=(x+y)∧u,x∗y=(x+y−u)∨0.

We note that MV-algebras provide a generalization of Boolean algebras in the sense that every Boolean algebra is an MV-algebra satisfying the condition x⊕x=x. For this reason, in order to generalize the concept of probability on Boolean algebras, Mundici introduced in [29] the notion of a state on an MV-algebra in the following way. Let A =(A,⊕,∗,0,u) be an MV-algebra. A mapping s:A→[0,1] is a state on A whenever s(0)=0 and, for every x,y∈A, the following condition is satisfied: if x∗y=0, then s(x⊕y)=s(x)+s(y). Since the definitions of product MV-algebra and partition in a product MV-algebra are based on the Mundici representation theorem (i.e., the MV-algebra operation ⊕ is substituted by the group operation + in the abelian *l*-group corresponding to the considered MV-algebra), in this contribution, we shall use the following (equivalent) definition of a state which is also based on the Mundici representation theorem. This means that the sum in the following definition of a state, and subsequently in what follows, denotes the sum in the abelian *l*-group that corresponds to the given MV-algebra.

**Definition** **2** ([27])**.** *A state on an MV-algebra* A=(A,⊕,∗,0,u) *is a mapping* s:A→[0,1] *with the following two properties:*

*(i)*  s(u)=1;
*(ii)*  *if*x,y∈A*such that*x+y≤u,*then*s(x+y)=s(x)+s(y).

**Definition** **3** ([19])**.** *A product MV-algebra is an algebraic structure* (A,⊕,∗,⋅,0,u), *where* (A,⊕,∗,0,u) *is an MV-algebra and* ⋅ *is an associative and abelian binary operation on A with the following properties:*

*(i)*  *for every*x∈A,u⋅x=x;
*(ii)*  *if*x,y,z∈A*such that*x+y≤u,*then*z⋅x+z⋅y≤u,*and*z⋅(x+y)=z⋅x+z⋅y.

For brevity, we will write (A,⋅) instead of (A,⊕,∗,⋅,0,u). A relevant probability theory for the product MV-algebras was developed by Riečan in [30], see also [31,32]; the entropy theory of Shannon and Kolmogorov-Sinai type for the product MV-algebras was proposed in [22,23]. We present the main idea and some results of these theories that will be used in the following text.

As in [22], by a partition in a product MV-algebra (A,⋅), we understand any *n*-tuple $X=(x_{1},x_{2},\ldots ,x_{n})$ of elements of *A* with the property $x_{1}+x_{2}+\ldots +x_{n}=u.$ In the system of all partitions in a given product MV-algebra (A,⋅), we define the refinement partial order ≻ in a standard way (cf. [33]). If $X=(x_{1},x_{2},\ldots ,x_{n}),$ and $Y=(y_{1},y_{2},\ldots ,y_{m})$ are two partitions in (A,⋅), then we write Y≻X (and we say that Y is a refinement of X), if there exists a partition {J(1),J(2),…,J(n)} of the set {1,2,…,m} such that $x_{i}=\sum_{j\in J(i)}y_{j},$ for i=1,2,…,n. Further, for two finite sequences $X=(x_{1},x_{2},\ldots ,x_{n}),$ and $Y=(y_{1},y_{2},\ldots ,y_{m})$ of elements of *A*, we put $X\vee Y=(x_{i}\cdot y_{j};i=1,2,\ldots ,n,$ j=1,2,…,m). If $X=(x_{1},x_{2},\ldots ,x_{n}),$ and $Y=(y_{1},y_{2},\ldots ,y_{m})$ are partitions in (A,⋅), then the system X∨Y is a partition in (A,⋅), since $\sum_{i=1}^{n}\sum_{j=1}^{m}x_{i}\cdot y_{j}$$=\left(\sum_{i=1}^{n}x_{i}\right)\cdot \left(\sum_{j=1}^{m}y_{j}\right)=u\cdot u=u.$ Later we shall need the following assertion:

**Proposition** **1.** *If* X,Y,Z *are partitions in a product MV-algebra* (A,⋅), *then it holds* X∨Y≻X, *and* Y≻X *implies* Y∨Z≻X∨Z.

**Proof.**  The proof can be found in [33]. ☐

The following example shows that the model studied in this article generalizes the classical case.

**Example** **1.** *Let us consider a probability space* (Ω,S,P) *and put* $A=\left\{I_{E};E\in S\right\},$ *where* $I_{E}:\Omega \to \left\{0,1\right\}$ *is the indicator function of the set* E∈S. *The class A is closed with respect to the product of indicator functions and it represents a special case of product MV-algebras. The mapping* s:A→[0,1] *defined, for every* $I_{E}\in A,$ *by* $s(I_{E})=P(E),$ *is a state on the considered product MV-algebra* (A,⋅). *A measurable partition* $\left\{E_{1},E_{2},\ldots ,E_{n}\right\}$ *of* (Ω,S,P) *can be viewed as a partition in the product MV-algebra* (A,⋅), *if we consider the n-tuple* $\left(I_{E_{1}},I_{E_{2}},\ldots ,I_{E_{n}}\right)$ *instead of* $\left\{E_{1},E_{2},\ldots ,E_{n}\right\}.$

**Example** **2.** *Let* (Ω,S,P) *be a probability space, and A be the class of all* S- *measurable functions* f:Ω→[0,1], *so called full tribe of fuzzy sets (cf., e.g., [21,34]). The class A is closed with respect to the natural product of fuzzy sets and it represents a significant case of product MV-algebras. The map* s:A→[0,1] *defined, for every* f∈A, *by* $s(f)=\int_{\Omega }fdP,$ *is a state on the product MV-algebra* (A,⋅). *The notion of a partition in the product MV-algebra* (A,⋅) *coincides with the notion of a fuzzy partition (cf. [34]).*

**Definition** **4.** *Let* s *be a state on a product MV-algebra* (A,⋅). *We say that partitions* X,Y *in* (A,⋅) *are statistically independent with respect to* s, *if* s(x⋅y)=s(x)⋅s(y), *for every* x∈X, *and* y∈Y.

The following definition of entropy of Shannon type was introduced in [22].

**Definition** **5.** *Let* $X=(x_{1},x_{2},\ldots ,x_{n})$ *be a partition in a product MV-algebra* (A,⋅) *and let* s:A→[0,1] *be a state. Then the entropy of* X *with respect to* s *is defined by Shannon’s formula*

(1) $$H_{s}(X)=-\sum_{i=1}^{n}s(x_{i})\cdot \log s(x_{i}).$$

*If* $X=(x_{1},x_{2},\ldots ,x_{n}),$ *and* $Y=(y_{1},y_{2},\ldots ,y_{m})$ *are two partitions in* (A,⋅), *then the conditional entropy of* X *given* $y_{j}\in Y$ *is defined by*

$$H_{s}(X/y_{j})=-\sum_{i=1}^{n}s(x_{i}/y_{j})\cdot \log s(x_{i}/y_{j}),$$

*where*

(2) $$s(x_{i}/y_{j})=\left\{ \begin{array}{ll} \frac{s(x_{i}\cdot y_{j})}{s(y_{j})}, & \mathrm{if}\;s(y_{j})>0;\\ 0, & \mathrm{if}\;s(y_{j})=0. \end{array}\right.$$

*The conditional entropy of* X *given* Y *is defined by*

(3) $$H_{s}(X/Y)=\sum_{j=1}^{m}s(y_{j})\cdot H_{s}(X/y_{j})=-\sum_{i=1}^{n}\sum_{j=1}^{m}s(x_{i}\cdot y_{j})\cdot \log\frac{s(x_{i}\cdot y_{j})}{s(y_{j})}.$$

Here, the usual convention that $0\log\frac{0}{x}=0$ if x≥0 is used. The base of the logarithm can be any positive real number, but as a rule one takes logarithms to the base 2. The entropy is then expressed in bits. The entropy and the conditional entropy of partitions in a product MV-algebra satisfy all properties that correspond to properties of Shannon’s entropy of measurable partitions in the classical case; for more details, see [22].

In [24], the concepts of mutual information and Kullback–Leibler divergence in a product MV-algebra were introduced in the following way.

**Definition** **6.** *Let* X,Y *be partitions in a product MV-algebra* (A,⋅), *and let* s:A→[0,1] *be a state. We define the mutual information between X and Y by the formula*

(4) $$I_{s}(X,Y)=H_{s}(X)-H_{s}(X/Y).$$

**Definition** **7.** *Let* s,t *be states defined on a given product MV-algebra* (A,⋅), *and* $X=(x_{1},x_{2},\ldots ,x_{n})$ *be a partition in* (A,⋅). *Then we define the* *Kullback–Leibler* *divergence* $D_{X}(s∥t)$ *by the formula*

(5) $$D_{X}(s∥t)=\sum_{i=1}^{n}s(x_{i})\cdot \log\frac{s(x_{i})}{t(x_{i})}.$$

The logarithm in this formula is taken to the base 2 and information is measured in units of bits. We use the convention that $x\log\frac{x}{0}=\infty$ if x>0, and $0\log\frac{0}{x}=0$ if x≥0.

In the proofs, we shall use the Jensen inequality which states that for a real concave function φ, real numbers $a_{1},a_{2},\ldots ,a_{n}$ in its domain and nonnegative real numbers $c_{1},c_{2},\ldots ,c_{n}$ such that $\sum_{i=1}^{n}c_{i}=1,$ it holds

(6) $$\varphi \left(\sum_{i=1}^{n}c_{i}a_{i}\right)\geq \sum_{i=1}^{n}c_{i}\varphi (a_{i}),$$

and the inequality is reversed if φ is a real convex function. The equality holds if and only if $a_{1}=a_{2}=\ldots =a_{n}$ or φ is linear.

Further, we recall the following notions.

**Definition** **8.** *Let D be a non-empty set and* f:D→ℜ *be a real function defined on it. Then the support of* f *is defined by supp* (f)={x∈D;f(x)≠0}.

**Definition** **9.** *Let* f:D→ℜ *be a real function defined on a non-empty set D. Define the* q *-norm, for* 1≤q<∞, *or* q *-quasinorm, for* 0<q<1, *of* f *as*

$$∥f{∥}_{q}={\left(\sum_{x\in D}{\left|f(x)\right|}^{q}\right)}^{\frac{1}{q}}.$$

## 3. The Rényi Entropy of a Partition in a Product MV-Algebra

In this section, we define the Rényi entropy of a partition in a product MV-algebra (A,⋅), and examine its properties. In the following, we assume that s:A→[0,1] is a state.

**Definition** **10.** *Let* $X=(x_{1},x_{2},\ldots ,x_{n})$ *be a partition in a product MV-algebra* (A,⋅). *Then we define the Rényi entropy of order* q, *where* q∈(0,1)∪(1,∞), *of the partition* X *with respect to* s *as the number:*

(7) $$H_{q}^{s}(X)=\frac{1}{1-q}\log\sum_{i=1}^{n}s{\left(x_{i}\right)}^{q}.$$

**Remark** **1.** *In accordance with the classical theory, the log is to the base 2 and the Rényi entropy is expressed in bits. For simplicity, we write* $s{\left(x_{i}\right)}^{q}$ *instead of* ${\left(s(x_{i})\right)}^{q}$ *and* $\log\sum_{i=1}^{n}s{\left(x_{i}\right)}^{q}$ *instead of* $\log\left(\sum_{i=1}^{n}s{\left(x_{i}\right)}^{q}\right).$

Let $X=(x_{1},x_{2},\ldots ,x_{n})$ be a partition in (A,⋅). If we consider the function $s_{X}:X\to ℜ,$ defined, for every $x_{i}\in X,$ by $s_{X}(x_{i})=s(x_{i}),$ then we have

$$∥s_{X}{∥}_{q}={\left(\sum_{i=1}^{n}s{\left(x_{i}\right)}^{q}\right)}^{\frac{1}{q}},$$

and the Formula (7) can be expressed in the following equivalent form:

(8) $$H_{q}^{s}(X)=\frac{q}{1-q}\log\left(∥s_{X}{∥}_{q}\right).$$

**Example** **3.** *Let* $X=(x_{1},x_{2},\ldots ,x_{n})$ *be any partition in a product MV-algebra* (A,⋅). *Let* s:A→[0,1] *be a uniform state over X, i.e.,* $s(x_{i})=\frac{1}{n},$ *for* i=1,2,…,n. *Then*

$$H_{q}^{s}(X)=\frac{1}{1-q}\log\sum_{i=1}^{n}{\left(\frac{1}{n}\right)}^{q}=\frac{1}{1-q}\log n^{1-q}=\log n.$$

**Example** **4.** *Let us consider any product MV-algebra*(A,⋅),*and the partition*E=(u)*in*(A,⋅)*that represents an experiment resulting in a certain event. It is easy to see that*$H_{q}^{s}(E)=0$.

**Remark** **2.** *It is possible to verify that the Rényi entropy*$H_{q}^{s}(X)$*is always nonnegative. Namely, for*q∈(0,1),*and*i=1,2,…,n,*we have*$s{\left(x_{i}\right)}^{q}\geq s(x_{i}),$*hence*$\sum_{i=1}^{n}s{\left(x_{i}\right)}^{q}\geq \sum_{i=1}^{n}s(x_{i})=s\left(\sum_{i=1}^{n}x_{i}\right)=s(u)=1$. *It follows that*$H_{q}^{s}(X)=\frac{1}{1-q}\log\sum_{i=1}^{n}s{\left(x_{i}\right)}^{q}\geq 0$. *On the other hand, for*q∈(1,∞),*and*i=1,2,…,n,*it holds*$s{\left(x_{i}\right)}^{q}\leq s(x_{i}),$*hence*$\sum_{i=1}^{n}s{\left(x_{i}\right)}^{q}\leq \sum_{i=1}^{n}s(x_{i})=1$. *The assumption*q∈(1,∞)*implies*$\frac{1}{1-q}<0,$*therefore, we get*$H_{q}^{s}(X)=\frac{1}{1-q}\log\sum_{i=1}^{n}s{\left(x_{i}\right)}^{q}\geq 0$.

At q=1 the value of the quantity $H_{q}^{s}(X)$ is undefined since it generates the form $\frac{0}{0}.$ In the following theorem, it is shown that for q→1 the Rényi entropy $H_{q}^{s}(X)$ converges to the Shannon entropy of a partition *X* in (A,⋅) defined by the formula (1).

**Theorem** **1.** *Let* $X=(x_{1},x_{2},\ldots ,x_{n})$ *be any partition in a product MV-algebra* (A,⋅). *Then*

$$\underset{q\to 1}{\lim}H_{q}^{s}(X)=-\sum_{i=1}^{n}s(x_{i})\cdot \log s(x_{i}).$$

**Proof.**  Put $f(q)=\log\sum_{i=1}^{n}s{\left(x_{i}\right)}^{q},$ and g(q)=1−q, for every q∈(0,∞). The functions f,g are differentiable and for every q∈(0,1)∪(1,∞), we have $H_{q}^{s}(X)=\frac{f(q)}{g(q)}.$ Evidently, $\underset{q\to 1}{\lim}g(q)=0,$ and $\underset{q\to 1}{\lim}f(q)=\underset{q\to 1}{\lim}\log\sum_{i=1}^{n}s{\left(x_{i}\right)}^{q}=\log\sum_{i=1}^{n}s(x_{i})=\log1=0$. Using L’Hôpital’s rule, this yields that $\underset{q\to 1}{\lim}H_{q}^{s}(X)=\underset{q\to 1}{\lim}\frac{f^{\prime }(q)}{g^{\prime }(q)},$ under the assumption that the right hand side exists. It holds $\frac{d}{dq}g(q)=-1,$ and

$$\begin{array}{c} \frac{d}{dq}f(q)=\frac{1}{(\ln2)\sum_{i=1}^{n}s{\left(x_{i}\right)}^{q}}\sum_{i=1}^{n}\frac{d}{dq}s{\left(x_{i}\right)}^{q}\\ =\frac{1}{(\ln2)\sum_{i=1}^{n}s{\left(x_{i}\right)}^{q}}\sum_{i=1}^{n}s{\left(x_{i}\right)}^{q}\ln s(x_{i})=\frac{1}{\sum_{i=1}^{n}s{\left(x_{i}\right)}^{q}}\sum_{i=1}^{n}s{\left(x_{i}\right)}^{q}\log s(x_{i}). \end{array}$$

Note that the calculation of the derivative of function *f* is easily done by using the identity $b^{\alpha }=e^{\alpha \ln b}.$ We get

$$\underset{q\to 1}{\lim}H_{q}^{s}(X)=\underset{q\to 1}{\lim}\frac{-1}{\sum_{i=1}^{n}s{\left(x_{i}\right)}^{q}}\sum_{i=1}^{n}s{\left(x_{i}\right)}^{q}\log s(x_{i})=-\sum_{i=1}^{n}s(x_{i})\log s(x_{i}),$$

which is the Shannon entropy of *X* defined by the Formula (1). ☐

In the following theorem it is proved that the function $H_{q}^{s}(X)$ is monotonically decreasing in q.

**Theorem** **2.** *Let X be any partition in a given product MV-algebra* (A,⋅), *and* $q_{1},q_{2}\in (0,1)\cup (1,\infty ).$ *Then* $q_{1}\geq q_{2}$ *implies* $H_{q_{1}}^{s}(X)\leq H_{q_{2}}^{s}(X).$

**Proof.**  Suppose that *X = x*_1_, *x*_2_, …, *x_n_* and $q_{1},q_{2}\in (1,\infty )$ such that $q_{1}\geq q_{2}.$ Then the claim is equivalent to the inequality

$${\left(\sum_{i=1}^{n}s{\left(x_{i}\right)}^{q_{1}}\right)}^{\frac{1}{q_{1}-1}}\geq {\left(\sum_{i=1}^{n}s{\left(x_{i}\right)}^{q_{2}}\right)}^{\frac{1}{q_{2}-1}}.$$

The above inequality follows by applying the Jensen inequality to the function φ, defined, for every x∈[0,∞), by $\varphi (x)=x^{\frac{q_{2}-1}{q_{1}-1}}.$ The assumption $q_{1}\geq q_{2}$ implies $\frac{q_{2}-1}{q_{1}-1}\leq 1,$ hence the function φ is concave. Putting $c_{i}=s(x_{i}),$ and $a_{i}=s{\left(x_{i}\right)}^{q_{1}-1},$ i=1,2,…,n, in the inequality (6), we get

$$\begin{array}{c} {\left(\sum_{i=1}^{n}s{\left(x_{i}\right)}^{q_{1}}\right)}^{\frac{1}{q_{1}-1}}={\left(\sum_{i=1}^{n}s(x_{i})s{\left(x_{i}\right)}^{q_{1}-1}\right)}^{\frac{q_{2}-1}{\left(q_{1}-1)(q_{2}-1\right)}}\\ ={\left({\left[\sum_{i=1}^{n}s(x_{i})s{\left(x_{i}\right)}^{q_{1}-1}\right]}^{\frac{q_{2}-1}{q_{1}-1}}\right)}^{\frac{1}{q_{2}-1}}\geq {\left(\sum_{i=1}^{n}s(x_{i})s{\left(x_{i}\right)}^{q_{2}-1}\right)}^{\frac{1}{q_{2}-1}}={\left(\sum_{i=1}^{n}s{\left(x_{i}\right)}^{q_{2}}\right)}^{\frac{1}{q_{2}-1}}. \end{array}$$

The case where $q_{1},q_{2}\in (0,1)$ is obtained in an analogous way. Finally, the case where $q_{1}\in (1,\infty ),$ and $q_{2}\in (0,1)$ is obtained by transitivity. ☐

**Example** **5.** *Consider any product MV-algebra* (A,⋅), *and a state* s:A→[0,1]. *Let* x∈A *with* s(x)=p, *where* p∈(0,1). *Then* s(u−x)=1−p, *and the pair* X=(x,u−x) *is a partition in* (A,⋅). *If we put* $p=\frac{1}{2},$ *then we have* $H_{q}^{s}(X)=1$ *bit, for every* q∈(0,1)∪(1,∞). *Put* $p=\frac{1}{3}.$ *By simple calculations we get* $H_{1/2}^{s}(X)\dot{=}0.958$ *bit,* $H_{2}^{s}(X)\dot{=}0.848$ *bit,* $H_{1/3}^{s}(X)\dot{=}0.972$ *bit. So, it holds* $H_{2}^{s}(X)<H_{1/2}^{s}(X)<H_{1/3}^{s}(X),$ *which is consistent with the property proven in the previous theorem.*

**Theorem** **3.** *Let* X,Y *be partitions in a product MV-algebra* (A,⋅) *such that* Y≻X. *Then* $H_{q}^{s}(X)\leq H_{q}^{s}(Y).$

**Proof.**  Suppose that $X=(x_{1},x_{2},\ldots ,x_{n}),$$Y=(y_{1},y_{2},\ldots ,y_{m}),$Y≻X. Then there exists a partition {J(1),J(2),…,J(n)} of the set {1,2,…,m} such that $x_{i}=\sum_{j\in J(i)}y_{j},$ for i=1,2,…,n. Hence $s(x_{i})=s\left(\sum_{j\in J(i)}y_{j}\right)=\sum_{j\in J(i)}s(y_{j}),$ for i=1,2,…,n.

(i) Consider the case when q∈(1,∞). Then $s{\left(x_{i}\right)}^{q}={\left(\sum_{j\in J(i)}s(y_{j})\right)}^{q}\geq \sum_{j\in J(i)}s(y_{j}{)}^{q},$ for i=1,2,…,n, and consequently
  $$\sum_{i=1}^{n}s{\left(x_{i}\right)}^{q}\geq \sum_{i=1}^{n}\sum_{j\in J(i)}s(y_{j}{)}^{q}=\sum_{j=1}^{m}s{\left(y_{j}\right)}^{q}.$$
  Therefore, we get
  $$\log\sum_{i=1}^{n}s{\left(x_{i}\right)}^{q}\geq \log\sum_{j=1}^{m}s{\left(y_{j}\right)}^{q}.$$
  In this case we have $\frac{1}{1-q}<0,$ hence
  $$H_{q}^{s}(X)=\frac{1}{1-q}\log\sum_{i=1}^{n}s{\left(x_{i}\right)}^{q}\leq \frac{1}{1-q}\log\sum_{j=1}^{m}s{\left(y_{j}\right)}^{q}=H_{q}^{s}(Y).$$
(ii) Consider the case when q∈(0,1). Then $s{\left(x_{i}\right)}^{q}={\left(\sum_{j\in J(i)}s(y_{j})\right)}^{q}\leq \sum_{j\in J(i)}s(y_{j}{)}^{q},$ for i=1,2,…,n, and consequently
  $$\sum_{i=1}^{n}s{\left(x_{i}\right)}^{q}\leq \sum_{j=1}^{m}s{\left(y_{j}\right)}^{q}.$$
  Therefore, we get
  $$\log\sum_{i=1}^{n}s{\left(x_{i}\right)}^{q}\leq \log\sum_{j=1}^{m}s{\left(y_{j}\right)}^{q}.$$
  In this case we have $\frac{1}{1-q}>0,$ hence
  $$H_{q}^{s}(X)=\frac{1}{1-q}\log\sum_{i=1}^{n}s{\left(x_{i}\right)}^{q}\leq \frac{1}{1-q}\log\sum_{j=1}^{m}s{\left(y_{j}\right)}^{q}=H_{q}^{s}(Y).$$

☐

As an immediate consequence of the previous theorem and Proposition 1, we obtain the following result.

**Corollary** **1.** *For every partition* X,Y *in a product MV-algebra* (A,⋅), *it holds*

(9) $$H_{q}^{s}(X\vee Y)\geq \max\left[H_{q}^{s}(X),H_{q}^{s}(Y)\right].$$

**Example** **6.** *Consider the measurable space*([0,1],ℬ),*where*ℬ*is the*σ-*algebra of all Borel subsets of the unit interval*[0,1].*Let A be the family of all Borel measurable functions*f:[0,1]→[0,1].*If we define in the family A the operation*⋅*as the natural product of fuzzy sets, then the system*(A,⋅)*is a product MV-algebra. We define a state*s:A→[0,1]*by the equality*$s(f)=\int_{0}^{1}f(x)dx,$*for any element*f*of A. It is easy to see that the pairs*$X=(f_{1},f_{2}),$$Y=(g_{1},g_{2}),$*where*$f_{1}(x)=x,$$f_{2}(x)=1-x,$$g_{1}(x)=x^{2},$$g_{2}(x)=1-x^{2},$x∈[0,1],*are partitions in*(A,⋅)*with the state values*$\frac{1}{2},\frac{1}{2}$*and*$\frac{1}{3},\frac{2}{3}$*of the corresponding elements, respectively. The join of partitions X and Y is the system*$X\vee Y=(f_{1}\cdot g_{1},f_{1}\cdot g_{2},f_{2}\cdot g_{1},f_{2}\cdot g_{2})$*with the state values*$\frac{1}{4},\frac{1}{4},\frac{1}{12},\frac{5}{12}$*of the corresponding elements*. *Using Formula (7), it can be computed that*$H_{1/2}^{s}(X\vee Y)\dot{=}1.903$*bit,*$H_{2}^{s}(X\vee Y)\dot{=}1.710$*bit. We have*$H_{1/2}^{s}(X)=H_{2}^{s}(X)=1$*bit,*$H_{1/2}^{s}(Y)\dot{=}0.958$*bit, and*$H_{2}^{s}(Y)\dot{=}0.848$*bit. It can be seen that the inequality (9) applies.*

**Theorem** **4.** *If partitions* X,Y *in a product MV-algebra* (A,⋅) *are statistically independent with respect to* s, *then* $H_{q}^{s}(X\vee Y)=H_{q}^{s}(X)+H_{q}^{s}(Y).$

**Proof.**  Suppose that $X=(x_{1},x_{2},\ldots ,x_{n}),$ $Y=(y_{1},y_{2},\ldots ,y_{m}).$ Let us calculate:

$$\begin{array}{c} H_{q}^{s}(X\vee Y)=\frac{1}{1-q}\log\sum_{i=1}^{n}\sum_{j=1}^{m}s{\left(x_{i}\cdot y_{j}\right)}^{q}=\frac{1}{1-q}\log\left(\sum_{i=1}^{n}s{\left(x_{i}\right)}^{q}\cdot \sum_{j=1}^{m}s{\left(y_{j}\right)}^{q}\right)\\ =\frac{1}{1-q}\log\sum_{i=1}^{n}s{\left(x_{i}\right)}^{q}+\frac{1}{1-q}\log\sum_{j=1}^{m}s{\left(y_{j}\right)}^{q}=H_{q}^{s}(X)+H_{q}^{s}(Y). \end{array}$$

☐

## 4. The Conditional Rényi Entropy in a Product MV-Algebra

In this section, we introduce the concept of conditional Rényi entropy $H_{q}^{s}(X/Y)$ of partitions in a product MV-algebra (A,⋅), analogously to [6]. It is shown that the proposed definition is consistent, in the case of the limit of *q* going to 1, with the conditional Shannon entropy defined by Equation (3). Subsequently, by using the proposed notion of conditional Rényi entropy, we define the Rényi information about a partition *X* in a partition *Y*.

Let $X=(x_{1},x_{2},\ldots ,x_{n}),$ and $Y=(y_{1},y_{2},\ldots ,y_{m})$ be two partitions in a product MV-algebra (A,⋅), and $y_{j}\in Y$ be fixed. If we consider the function $s_{X/y_{j}}:X\to ℜ,$ defined, for every $x_{i}\in X,$ by $s_{X/y_{j}}(x_{i})=s(x_{i}/y_{j}),$ then we have

$$∥s_{X/y_{j}}{∥}_{q}={\left(\sum_{i=1}^{n}s{\left(x_{i}/y_{j}\right)}^{q}\right)}^{\frac{1}{q}}.$$

**Definition** **11.** *Let* $X=(x_{1},x_{2},\ldots ,x_{n}),$ *and* $Y=(y_{1},y_{2},\ldots ,y_{m})$ *be partitions in* (A,⋅). *We define the conditional Rényi entropy of order* q, *where* q∈(0,1)∪(1,∞), *of X given Y by the formula*

(10) $$H_{q}^{s}(X/Y)=\frac{q}{1-q}\log\left(\sum_{j=1}^{m}s(y_{j})∥s_{X/y_{j}}{∥}_{q}\right).$$

**Remark** **3.** *In the same way as in the unconditional case, it can be verified that the conditional Rényi entropy* $H_{q}^{s}(X/Y)$ *is always nonnegative. Let* $X=(x_{1},x_{2},\ldots ,x_{n})$ *be any partition in* (A,⋅), *and* E=(u). *Since* $s_{X/u}(x_{i})=s(x_{i}/u)=s(x_{i})=s_{X}(x_{i}),$ *for* i=1,2,…,n, *it holds* $∥s_{X/u}{∥}_{q}=∥s_{X}{∥}_{q},$ *and consequently*

$$H_{q}^{s}(X/E)=\frac{q}{1-q}\log\left(s(u)∥s_{X/u}{∥}_{q}\right)=\frac{q}{1-q}\log\left(∥s_{X}{∥}_{q}\right)=H_{q}^{s}(X).$$

**Proposition** **2.** *Let* $X=(x_{1},x_{2},\ldots ,x_{n})$ *be a partition in a product MV-algebra* (A,⋅) *and let* s:A→[0,1] *be a state. Then:*

*(i)*  $\sum_{i=1}^{n}s(x_{i}\cdot y)=s(y),$ *for any element* y∈A;
*(ii)*  $\sum_{i=1}^{n}s(x_{i}/y)=1,$*for any element*y∈A*such that*s(y)>0.

**Proof.**  The proof of the claim (i) can be found in [33]. If y∈A such that s(y)>0, then using the previous equality, we get

$$\sum_{i=1}^{n}s(x_{i}/y)=\frac{1}{s(y)}\sum_{i=1}^{n}s(x_{i}\cdot y)=\frac{s(y)}{s(y)}=1.$$

☐

**Theorem** **5.** *Let* $X=(x_{1},x_{2},\ldots ,x_{n}),$ *and* $Y=(y_{1},y_{2},\ldots ,y_{m})$ *be partitions in* (A,⋅). *Then*

$$\underset{q\to 1}{\lim}H_{q}^{s}(X/Y)=-\sum_{i=1}^{n}\sum_{j=1}^{m}s(x_{i}\cdot y_{j})\cdot \log\frac{s(x_{i}\cdot y_{j})}{s(y_{j})}.$$

**Proof.**  For every q∈(0,1)∪(1,∞), we have

$$H_{q}^{s}(X/Y)=\frac{q}{1-q}\log\left(\sum_{j=1}^{m}s(y_{j})∥s_{X/y_{j}}{∥}_{q}\right)=-\frac{1}{1-\frac{1}{q}}\log\left(\sum_{j=1}^{m}s(y_{j})∥s_{X/y_{j}}{∥}_{q}\right)=-\frac{f(q)}{g(q)},$$

where f and g are continuous functions defined, for every q∈(0,∞), by the equalities

$$f(q)=\log\left(\sum_{j=1}^{m}s(y_{j})∥s_{X/y_{j}}{∥}_{q}\right),\;g(q)=1-\frac{1}{q}.$$

The functions f and g are differentiable and evidently, $\underset{q\to 1}{\lim}g(q)=0$. Also, it can easily be verified that $\underset{q\to 1}{\lim}f(q)=0$. Indeed, if we put $\delta =\left\{j;s(y_{j})>0\right\},$ then using Proposition 2, we get

$$\begin{array}{c} \underset{q\to 1}{\lim}f(q)=\log\left(\sum_{j=1}^{m}s(y_{j})\sum_{i=1}^{n}s(x_{i}/y_{j})\right)=\log\left(\sum_{j\in \delta }s(y_{j})\sum_{i=1}^{n}s(x_{i}/y_{j})\right)\\ =\log\left(\sum_{j\in \delta }s(y_{j})\sum_{i=1}^{n}\frac{s(x_{i}\cdot y_{j})}{s(y_{j})}\right)=\log\left(\sum_{j\in \delta }\sum_{i=1}^{n}s(x_{i}\cdot y_{j})\right)\\ =\log\left(\sum_{j\in \delta }s(y_{j})\right)=\log\left(\sum_{j=1}^{m}s(y_{j})\right)=\log1=0. \end{array}$$

Using L’Hôpital’s rule, it follows that $\underset{q\to 1}{\lim}H_{q}^{s}(X/Y)=-\underset{q\to 1}{\lim}\frac{f^{\prime }(q)}{g^{\prime }(q)},$ under the assumption that the right-hand side exists. Let us calculate the derivatives of the functions *f* and *g*. We have $\frac{d}{dq}g(q)=\frac{1}{q^{2}},$ and $\frac{d}{dq}f(q)=\frac{h^{\prime }(q)}{h(q)\ln2},$ where h(q) is the continuous function defined, for every q∈(0,∞), by the formula $h(q)=\sum_{j=1}^{m}s(y_{j})∥s_{X/y_{j}}{∥}_{q}$ with the continuous derivative $h^{\prime }$ for which it holds

$$h^{\prime }(q)=\sum_{j=1}^{m}s(y_{j})∥s_{X/y_{j}}{∥}_{q}\cdot \left[-\frac{1}{q^{2}}\ln\sum_{i=1}^{n}s{\left(x_{i}/y_{j}\right)}^{q}+\frac{1}{q}\frac{1}{\sum_{i=1}^{n}s{\left(x_{i}/y_{j}\right)}^{q}}\sum_{i=1}^{n}s{\left(x_{i}/y_{j}\right)}^{q}\ln s(x_{i}/y_{j})\right].$$

Analogously as in the proof of Theorem 1, we used the identity $b^{\alpha }=e^{\alpha \ln b}$ to calculate the derivative of function *h*.

We get

$$\underset{q\to 1}{\lim}f^{\prime }(q)=\frac{1}{\ln2}\sum_{j=1}^{m}s(y_{j})\sum_{i=1}^{n}s(x_{i}/y_{j})\ln s(x_{i}/y_{j})=\sum_{j=1}^{m}\sum_{i=1}^{n}s(x_{i}\cdot y_{j})\log\frac{s(x_{i}\cdot y_{j})}{s(y_{j})}.$$

It follows

$$\underset{q\to 1}{\lim}H_{q}^{s}(X/Y)=\underset{q\to 1}{-\lim}f^{\prime }(q)=-\sum_{i=1}^{n}\sum_{j=1}^{m}s(x_{i}\cdot y_{j})\cdot \log\frac{s(x_{i}\cdot y_{j})}{s(y_{j})},$$

which is the conditional Shannon entropy of *X* given *Y* defined by Equation (3). ☐

**Theorem** **6** (monotonicity)**.** *Let X and Y be partitions in a product MV-algebra* (A,⋅). *Then*

$$H_{q}^{s}(X/Y)\leq H_{q}^{s}(X).$$

**Proof.**  Let $X=(x_{1},x_{2},\ldots ,x_{n}),$ $Y=(y_{1},y_{2},\ldots ,y_{m}).$ Then by Proposition 2, it holds $s(x_{i})=\sum_{j=1}^{m}s(x_{i}\cdot y_{j}),$ for i=1,2,…,n. Suppose that q∈(1,∞). Then, using the triangle inequality of the q-norm, we get

$$\begin{array}{l} \sum_{i=1}^{n}s{\left(x_{i}\right)}^{q}=\sum_{i=1}^{n}{\left(\sum_{j=1}^{m}s(x_{i}\cdot y_{j})\right)}^{q}={\left({\left(\sum_{i=1}^{n}{\left(\sum_{j=1}^{m}s(x_{i}\cdot y_{j})\right)}^{q}\right)}^{\frac{1}{q}}\right)}^{q}\\ ={\left(∥\sum_{j=1}^{m}s_{X\vee (y_{j})}{∥}_{q}\right)}^{q}\leq {\left(\sum_{j=1}^{m}∥s_{X\vee (y_{j})}{∥}_{q}\right)}^{q}={\left(\sum_{j=1}^{m}s(y_{j})∥s_{X/y_{j}}{∥}_{q}\right)}^{q}. \end{array}$$

It follows that

$$\log\sum_{i=1}^{n}s{\left(x_{i}\right)}^{q}\leq \log{\left(\sum_{j=1}^{m}s(y_{j})∥s_{X/y_{j}}{∥}_{q}\right)}^{q},$$

and consequently

$$\begin{array}{ll} H_{q}^{s}(X)= & \frac{1}{1-q}\log\sum_{i=1}^{n}s{\left(x_{i}\right)}^{q}\geq \frac{1}{1-q}\log{\left(\sum_{j=1}^{m}s(y_{j})∥s_{X/y_{j}}{∥}_{q}\right)}^{q}\\ & =\frac{q}{1-q}\log\left(\sum_{j=1}^{m}s(y_{j})∥s_{X/y_{j}}{∥}_{q}\right)=H_{q}^{s}(X/Y). \end{array}$$

For the case where q∈(0,1), we put $r=\frac{1}{q}.$ By writing the Rényi entropy in terms of the $\frac{1}{q}$-norm and using the triangle inequality for the $\frac{1}{q}$-norm, we get

$$\begin{array}{c} H_{q}^{s}(X)=\frac{1}{1-q}\log\sum_{i=1}^{n}s{\left(x_{i}\right)}^{q}=\frac{r}{r-1}\log\sum_{i=1}^{n}s{\left(x_{i}\right)}^{\frac{1}{r}}=\frac{r}{r-1}\log\sum_{i=1}^{n}{\left(\sum_{j=1}^{m}s(x_{i}\cdot y_{j})\right)}^{\frac{1}{r}}\\ =\frac{r}{r-1}\log\sum_{i=1}^{n}∥s_{(x_{i})\vee Y}^{\frac{1}{r}}{∥}_{r}\geq \frac{r}{r-1}\log∥\sum_{i=1}^{n}s_{(x_{i})\vee Y}^{\frac{1}{r}}{∥}_{r}\\ =\frac{r}{r-1}\log{\left(\sum_{j=1}^{m}{\left(\sum_{i=1}^{n}s(x_{i}\cdot y_{j}{)}^{\frac{1}{r}}\right)}^{r}\right)}^{\frac{1}{r}}=\frac{r}{r-1}\log{\left(\sum_{j=1}^{m}s(y_{j}){\left(\sum_{i=1}^{n}s(x_{i}/y_{j}{)}^{\frac{1}{r}}\right)}^{r}\right)}^{\frac{1}{r}}\\ =\frac{1}{r-1}\log\left(\sum_{j=1}^{m}s(y_{j}){\left(\sum_{i=1}^{n}s(x_{i}/y_{j}{)}^{\frac{1}{r}}\right)}^{r}\right)=\frac{q}{1-q}\log\left(\sum_{j=1}^{m}s(y_{j})∥s_{X/y_{j}}{∥}_{q}\right)=H_{q}^{s}(X/Y). \end{array}$$

☐

**Theorem** **7.** *If partitions* X,Y *in a product MV-algebra* (A,⋅) *are statistically independent with respect to* s, *then* $H_{q}^{s}(X/Y)=H_{q}^{s}(X).$

**Proof.**  Suppose that $X=(x_{1},x_{2},\ldots ,x_{n}),$ $Y=(y_{1},y_{2},\ldots ,y_{m}),$ and put $\delta =\left\{j;s(y_{j})>0\right\}.$ Since it holds $\sum_{j\in \delta }s(y_{j})=\sum_{j=1}^{m}s(y_{j})=1,$ we get

$$\begin{array}{c} H_{q}^{s}(X/Y)=\frac{q}{1-q}\log\left(\sum_{j=1}^{m}s(y_{j}){\left(\sum_{i=1}^{n}s{\left(x_{i}/y_{j}\right)}^{q}\right)}^{\frac{1}{q}}\right)=\frac{q}{1-q}\log\left(\sum_{j\in \delta }s(y_{j}){\left(\sum_{i=1}^{n}\frac{s{\left(x_{i}\right)}^{q}s{\left(y_{j}\right)}^{q}}{s{\left(y_{j}\right)}^{q}}\right)}^{\frac{1}{q}}\right)\\ =\frac{q}{1-q}\log\left(\sum_{j\in \delta }s(y_{j}){\left(\sum_{i=1}^{n}s{\left(x_{i}\right)}^{q}\right)}^{\frac{1}{q}}\right)=\frac{1}{1-q}\log\sum_{i=1}^{n}s{\left(x_{i}\right)}^{q}=H_{q}^{s}(X). \end{array}$$

☐

**Theorem** **8.** *Let X, Y be partitions in a product MV-algebra* (A,⋅), *and* $q_{1},q_{2}\in (0,1)\cup (1,\infty ).$ *Then* $q_{1}\geq q_{2}$ *implies* $H_{q_{1}}^{s}(X/Y)\leq H_{q_{2}}^{s}(X/Y).$

**Proof.**  Suppose that $X=(x_{1},x_{2},\ldots ,x_{n}),$ $Y=(y_{1},y_{2},\ldots ,y_{m}),$ and $q_{1},q_{2}\in (1,\infty ).$ Then the claim is equivalent to the inequality

(11) $${\left(\sum_{j=1}^{m}s(y_{j})∥s_{X/y_{j}}{∥}_{q_{1}}\right)}^{\frac{q_{1}}{q_{1}-1}}\geq {\left(\sum_{j=1}^{m}s(y_{j})∥s_{X/y_{j}}{∥}_{q_{2}}\right)}^{\frac{q_{2}}{q_{2}-1}}.$$

We prove this inequality by applying twice the Jensen inequality. First, we apply the Jensen inequality to the function $\varphi_{1}$ defined, for every x∈[0,∞), by $\varphi_{1}(x)=x^{\frac{q_{1}(q_{2}-1)}{q_{2}(q_{1}-1)}}.$ The assumption $q_{1}\geq q_{2}$ implies that $\frac{q_{1}(q_{2}-1)}{q_{2}(q_{1}-1)}\leq 1,$ hence the function $\varphi_{1}$ is concave. Therefore, if we put $c_{j}=s(y_{j}),$ and $a_{j}=∥s_{X/y_{j}}{∥}_{q_{1}},$ j=1,2,…,m, in the inequality (6), we obtain

$$\begin{array}{c} {\left(\sum_{j=1}^{m}s(y_{j})∥s_{X/y_{j}}{∥}_{q_{1}}\right)}^{\frac{q_{1}}{q_{1}-1}}={\left[{\left(\sum_{j=1}^{m}s(y_{j})∥s_{X/y_{j}}{∥}_{q_{1}}\right)}^{\frac{q_{1}(q_{2}-1)}{q_{2}(q_{1}-1)}}\right]}^{\frac{q_{2}}{q_{2}-1}}\\ \geq {\left[\sum_{j=1}^{m}s(y_{j}){\left(∥s_{X/y_{j}}{∥}_{q_{1}}\right)}^{\frac{q_{1}(q_{2}-1)}{q_{2}(q_{1}-1)}}\right]}^{\frac{q_{2}}{q_{2}-1}}={\left[\sum_{j=1}^{m}s(y_{j}){\left(\sum_{i=1}^{n}s(x_{i}/y_{j})s{\left(x_{i}/y_{j}\right)}^{q_{1}-1}\right)}^{\frac{q_{2}-1}{q_{2}(q_{1}-1)}}\right]}^{\frac{q_{2}}{q_{2}-1}}. \end{array}$$

Next, we apply the Jensen inequality to the function $\varphi_{2}$ defined, for every x∈[0,∞), by $\varphi_{2}(x)=x^{\frac{q_{2}-1}{q_{1}-1}}.$ The assumption $q_{1}\geq q_{2}$ implies $\frac{q_{2}-1}{q_{1}-1}\leq 1,$ hence the function $\varphi_{2}$ is concave. Put $c_{i}=s(x_{i}/y_{j}),$ and $a_{i}=s{\left(x_{i}/y_{j}\right)}^{q_{1}-1},$ i=1,2,…,n, in the inequality (6). Note that according to Proposition 2, it holds $\sum_{i=1}^{n}c_{i}=1$. By the Jensen inequality we get

$$\begin{array}{c} {\left[\sum_{j=1}^{m}s(y_{j}){\left(\sum_{i=1}^{n}s(x_{i}/y_{j})s{\left(x_{i}/y_{j}\right)}^{q_{1}-1}\right)}^{\frac{q_{2}-1}{q_{2}(q_{1}-1)}}\right]}^{\frac{q_{2}}{q_{2}-1}}\\ \geq {\left[\sum_{j=1}^{m}s(y_{j}){\left(\sum_{i=1}^{n}s{\left(x_{i}/y_{j}\right)}^{q_{2}}\right)}^{\frac{1}{q_{2}}}\right]}^{\frac{q_{2}}{q_{2}-1}}=\;{\left(\sum_{j=1}^{m}s(y_{j})∥s_{X/y_{j}}{∥}_{q_{2}}\right)}^{\frac{q_{2}}{q_{2}-1}}. \end{array}$$

By combining the previous results, we obtain the inequality (11). Analogously, we can prove the inequality for the case where $q_{1},q_{2}\in (0,1).$ Finally, the case where $q_{1}\in (1,\infty )$ and $q_{2}\in (0,1)$ follows by transitivity. ☐

In the following theorem, a weak chain rule for the Rényi entropy of partitions in a product MV-algebra (A,⋅) is given.

**Theorem** **9.** *Let* $X=(x_{1},x_{2},\ldots ,x_{n}),$ *and* $Y=(y_{1},y_{2},\ldots ,y_{m})$ *be partitions in a product MV-algebra* (A,⋅). *Then*

$$H_{q}^{s}(X\vee Y)\leq H_{q}^{s}(X/Y)+\log\beta ,$$

*where* $\beta =\max\left\{\frac{1}{s(y_{j})};y_{j}\in supp(s),j=1,2,\ldots ,m\right\}.$

**Proof.**  Put $\delta =\left\{j;s(y_{j})>0\right\}.$ The assertion follows by applying the Jensen inequality to the function φ defined by $\varphi (x)=x^{q},$ x∈[0,∞), and putting $c_{j}=s(y_{j}),$$a_{j}=∥s_{X/y_{j}}{∥}_{q},$j=1,2,…,m.

Let q∈(0,1). Then the function φ is concave, and therefore we get

$$\begin{array}{c} {\left(\sum_{j=1}^{m}s(y_{j})∥s_{X/y_{j}}{∥}_{q}\right)}^{q}\geq {\sum_{j=1}^{m}s(y_{j})\left(∥s_{X/y_{j}}{∥}_{q}\right)}^{q}\\ =\sum_{j\in \delta }s(y_{j})\sum_{i=1}^{n}\frac{s{\left(x_{i}\cdot y_{j}\right)}^{q}}{s{\left(y_{j}\right)}^{q}}=\sum_{j\in \delta }{\left(\frac{1}{s(y_{j})}\right)}^{q-1}\sum_{i=1}^{n}s{\left(x_{i}\cdot y_{j}\right)}^{q}\geq \sum_{j\in \delta }\beta^{q-1}\sum_{i=1}^{n}s{\left(x_{i}\cdot y_{j}\right)}^{q}. \end{array}$$

It follows

$$\begin{array}{c} H_{q}^{s}(X/Y)=\frac{q}{1-q}\log\left(\sum_{j=1}^{m}s(y_{j})∥s_{X/y_{j}}{∥}_{q}\right)\geq \frac{1}{1-q}\log\left(\beta^{q-1}\sum_{j\in \delta }\sum_{i=1}^{n}s{\left(x_{i}\cdot y_{j}\right)}^{q}\right)\\ =-\log\beta +\frac{1}{1-q}\log\sum_{j=1}^{m}\sum_{i=1}^{n}s{\left(x_{i}\cdot y_{j}\right)}^{q}=-\log\beta +H_{q}^{s}(X\vee Y). \end{array}$$

Consider now the case where q∈(1,∞). Then the function φ is convex, and therefore we have

$$\begin{array}{ll} & {\left(\sum_{j=1}^{m}s(y_{j})∥s_{X/y_{j}}{∥}_{q}\right)}^{q}\leq {\sum_{j=1}^{m}s(y_{j})\left(∥s_{X/y_{j}}{∥}_{q}\right)}^{q}\\ = & \sum_{j\in \delta }{\left(\frac{1}{s(y_{j})}\right)}^{q-1}\sum_{i=1}^{n}s{\left(x_{i}\cdot y_{j}\right)}^{q}\leq \sum_{j\in \delta }\beta^{q-1}\sum_{i=1}^{n}s{\left(x_{i}\cdot y_{j}\right)}^{q}. \end{array}$$

Thus

$$q\log\left(\sum_{j=1}^{m}s(y_{j})∥s_{X/y_{j}}{∥}_{q}\right)\leq (q-1)\log\beta +\log\sum_{j=1}^{m}\sum_{i=1}^{n}s{\left(x_{i}\cdot y_{j}\right)}^{q}.$$

Since 1−q<0, for q∈(1,∞), we get

$$\begin{array}{c} H_{q}^{s}(X/Y)=\frac{q}{1-q}\log\left(\sum_{j=1}^{m}s(y_{j})∥s_{X/y_{j}}{∥}_{q}\right)\\ \geq \frac{q-1}{1-q}\log\beta +\frac{1}{1-q}\log\sum_{j=1}^{m}\sum_{i=1}^{n}s{\left(x_{i}\cdot y_{j}\right)}^{q}=-\log\beta +H_{q}^{s}(X\vee Y). \end{array}$$

☐

**Remark** **4.** *Let* $X=(x_{1},x_{2},\ldots ,x_{n}),$ *and* $Y=(y_{1},y_{2},\ldots ,y_{m})$ *be partitions in* (A,⋅). *Since* X∨Y=Y∨X, *it holds also the inequality* $H_{q}^{s}(X\vee Y)\leq H_{q}^{s}(Y/X)+\log\alpha ,$ *where* $\alpha =\max\left\{\frac{1}{s(x_{i})};x_{i}\in supp(s),i=1,2,\ldots ,n\right\}.$

**Corollary** **2.** *Let* $X=(x_{1},x_{2},\ldots ,x_{n}),$ *and* $Y=(y_{1},y_{2},\ldots ,y_{m})$ *be partitions in* (A,⋅). *Then:*

*(i)*  $H_{q}^{s}(X\vee Y)\leq H_{q}^{s}(X)+\log\beta ;$
*(ii)*  $H_{q}^{s}(X\vee Y)\leq H_{q}^{s}(Y)+\log\alpha ,$

*where* $\beta =\max\left\{\frac{1}{s(y_{j})};y_{j}\in supp(s),j=1,2,\ldots ,m\right\},$ *and* $\alpha =\max\left\{\frac{1}{s(x_{i})};x_{i}\in supp(s),i=1,2,\ldots ,n\right\}.$

**Proof.**  The claim is a direct consequence of Theorems 6 and 9. ☐

**Definition** **12.** *Let* X,Y *be partitions in* (A,⋅). *We define the Rényi information of order* q, *where* q∈(0,1)∪(1,∞), *about X in Y by the formula*

(12) $$I_{q}^{s}(X,Y)=H_{q}^{s}(X)-H_{q}^{s}(X/Y).$$

**Theorem** **10.** *Let* X,Y *be partitions in* (A,⋅). *Then* $\underset{q\to 1}{\lim}I_{q}^{s}(X,Y)=I_{s}(X,Y),$ *where* $I_{s}(X,Y)$ *is the mutual information of partitions* X,Y *defined by Equation (4).*

**Proof.**  The claim is obtained as a direct consequence of Theorems 1 and 5. ☐

**Theorem** **11.** *For arbitrary partitions*X,Y*in*(A,⋅),*it holds*$I_{q}^{s}(X,Y)\geq 0$. *Moreover, if*X,Y*are statistically independent with respect to*s,*then*$I_{q}^{s}(X,Y)=0$.

**Proof.**  The claim is a direct consequence of Theorems 6 and 7. ☐

## 5. The Rényi Divergence in a Product MV-Algebra

In this section, we introduce the concept of the Rényi divergence in a product MV-algebra (A,⋅). We will prove basic properties of this quantity, and for illustration, we provide some numerical examples.

**Definition** **13.** *Let* s,t *be states on a given product MV-algebra* (A,⋅), *and* $X=(x_{1},x_{2},\ldots ,x_{n})$ *be a partition in* (A,⋅) *such that* $t(x_{i})>0,$ *for* i=1,2,…,n. *Then we define the Rényi divergence* $D_{q}^{X}(s∥t)$ *of order* q, *where* q∈(0,1)∪(1,∞), *with respect to X as the number*

(13) $$D_{q}^{X}(s∥t)=\frac{1}{q-1}\log\sum_{i=1}^{n}s{\left(x_{i}\right)}^{q}t{\left(x_{i}\right)}^{1-q}.$$

**Remark** **5.** *The logarithm in the Formula (13) is taken to the base 2 and information is measured in bits. It is easy to see that*$D_{q}^{X}(s∥s)=0$. *Namely,*

$$D_{q}^{X}(s∥s)=\frac{1}{q-1}\log\sum_{i=1}^{n}s{\left(x_{i}\right)}^{q}s{\left(x_{i}\right)}^{1-q}=\frac{1}{q-1}\log\sum_{i=1}^{n}s(x_{i})=\frac{1}{q-1}\log1=0.$$

The following theorem states that for q→1 the Rényi divergence $D_{q}^{X}(s∥t)$ converges to the Kullback–Leibler divergence $D_{X}(s∥t)$ defined by the Formula (5).

**Theorem** **12.** *Let* s,t *be states on a given product MV-algebra* (A,⋅), *and* $X=(x_{1},x_{2},\ldots ,x_{n})$ *be a partition in* (A,⋅) *such that* $t(x_{i})>0,$ *for* i=1,2,…,n. *Then*

$$\underset{q\to 1}{\lim}D_{q}^{X}(s∥t)=\sum_{i=1}^{n}s(x_{i})\cdot \log\frac{s(x_{i})}{t(x_{i})}.$$

**Proof.**  For every q∈(0,1)∪(1,∞), we have

$$D_{q}^{X}(s∥t)=\frac{1}{q-1}\log\sum_{i=1}^{n}s{\left(x_{i}\right)}^{q}t{\left(x_{i}\right)}^{1-q}=\frac{f(q)}{g(q)},$$

where f,g are continuous functions defined, for every q∈(0,∞), in the following way:

$$f(q)=\log\sum_{i=1}^{n}s{\left(x_{i}\right)}^{q}t{\left(x_{i}\right)}^{1-q},\;g(q)=q-1.$$

By continuity of the functions f,g, we have $\underset{q\to 1}{\lim}f(q)=f(1)=\log\sum_{i=1}^{n}s(x_{i})t{\left(x_{i}\right)}^{0}=\log1=0,$ and $\underset{q\to 1}{\lim}g(q)=g(1)=0$. Using L’Hôpital’s rule, we get that $\underset{q\to 1}{\lim}D_{q}^{X}(s∥t)=\underset{q\to 1}{\lim}\frac{f^{\prime }(q)}{g^{\prime }(q)},$ under the assumption that the right hand side exists. Since $g^{\prime }(q)=1,$ and $f^{\prime }(q)=\frac{h^{\prime }(q)}{h(q)\ln2},$ where

$$h(q)=\sum_{i=1}^{n}s{\left(x_{i}\right)}^{q}t{\left(x_{i}\right)}^{1-q},\;h^{\prime }(q)=\sum_{i=1}^{n}s{\left(x_{i}\right)}^{q}t{\left(x_{i}\right)}^{1-q}\ln\frac{s(x_{i})}{t(x_{i})},$$

we obtain

$$\underset{q\to 1}{\lim}D_{q}^{X}(s∥t)=\underset{q\to 1}{\lim}f^{\prime }(q)=\frac{1}{\ln2}\sum_{i=1}^{n}s(x_{i})\ln\frac{s(x_{i})}{t(x_{i})}=\sum_{i=1}^{n}s(x_{i})\log\frac{s(x_{i})}{t(x_{i})}.$$

☐

The following theorem states that the Rényi entropy $H_{q}^{s}(X)$ can be expressed in terms of the Rényi divergence $D_{q}^{X}(s∥t)$ of a state *s* from a state *t* that is uniform over *X*.

**Theorem** **13.** *Let* s *be a state on a product MV-algebra* (A,⋅), *and* $X=(x_{1},x_{2},\ldots ,x_{n})$ *be a partition in* (A,⋅). *If a state* t:A→[0,1] *is uniform over X, i.e.,* $t(x_{i})=\frac{1}{n},$ *for* i=1,2,…,n, *then*

$$H_{q}^{s}(X)=H_{q}^{t}(X)-D_{q}^{X}(s∥t)=\log n-D_{q}^{X}(s∥t).$$

**Proof.**  Let us calculate:

$$\begin{array}{c} D_{q}^{X}(s∥t)=\frac{1}{q-1}\log\sum_{i=1}^{n}s{\left(x_{i}\right)}^{q}t{\left(x_{i}\right)}^{1-q}=\frac{1}{q-1}\log\sum_{i=1}^{n}s{\left(x_{i}\right)}^{q}{\left(\frac{1}{n}\right)}^{1-q}\\ =\frac{1}{q-1}\log{\left(\frac{1}{n}\right)}^{1-q}+\frac{1}{q-1}\log\sum_{i=1}^{n}s{\left(x_{i}\right)}^{q}=\log n-H_{q}^{s}(X). \end{array}$$

On the other hand, as shown by Example 3, it holds $H_{q}^{t}(X)=\log n.$ ☐

**Example** **7.** *Consider any product MV-algebra* (A,⋅), *and a state* s:A→[0,1]. *In Example 5, we dealt with the partition* X=(x,u−x) *with* $s(x)=\frac{1}{3},$ *and we calculated the Rényi entropy* $H_{1/2}^{s}(X)\dot{=}0.958$ *bit. Let* t:A→[0,1] *be a state uniform over X, i.e.,* $t(x)=t(u-x)=\frac{1}{2}.$ *Then the Rényi* *divergence* $D_{q}^{X}(s∥t)$ *of order* $q=\frac{1}{2}$ *is* $D_{1/2}^{X}(s∥t)=-2\log\left(\sqrt{\frac{1}{3}\cdot \frac{1}{2}}+\sqrt{\frac{2}{3}\cdot \frac{1}{2}}\right)\dot{=}0.04186$ *bit, and* $H_{1/2}^{t}(X)-D_{1/2}^{X}(s∥t)\dot{=}1-0.04186\dot{=}0.958$ *bit. So, the equality* $H_{1/2}^{s}(X)=H_{1/2}^{t}(X)-D_{1/2}^{X}(s∥t)$ *holds.*

**Theorem** **14.** *Let* s,t *be states on a given product MV-algebra* (A,⋅), *and* $X=(x_{1},x_{2},\ldots ,x_{n})$ *be a partition in* (A,⋅) *such that* $s(x_{i})>0,$ *and* $t(x_{i})>0,$ *for* i=1,2,…,n. *Then* $D_{q}^{X}(s∥t)\geq 0$ *with the equality if and only if* $s(x_{i})=t(x_{i}),$ *for* i=1,2,…,n.

**Proof.**  The inequality follows by applying the Jensen inequality to the function φ defined by $\varphi (x)=x^{1-q},$ x∈[0,∞), and putting $c_{i}=s(x_{i}),$ $a_{i}=\frac{t(x_{i})}{s(x_{i})},$ for i=1,2,…,n.

Let us consider the case of q∈(1,∞). Then 1−q<0, therefore the function φ is convex. By the Jensen inequality we obtain

(14) $$1={\left(\sum_{i=1}^{n}t(x_{i})\right)}^{1-q}={\left(\sum_{i=1}^{n}s(x_{i})\frac{t(x_{i})}{s(x_{i})}\right)}^{1-q}\leq {\sum_{i=1}^{n}s(x_{i})\left(\frac{t(x_{i})}{s(x_{i})}\right)}^{1-q}=\sum_{i=1}^{n}s{\left(x_{i}\right)}^{q}t{\left(x_{i}\right)}^{1-q},$$

and consequently

$$\log\sum_{i=1}^{n}s{\left(x_{i}\right)}^{q}t{\left(x_{i}\right)}^{1-q}\geq \log1=0.$$

Since $\frac{1}{q-1}>0,$ for q∈(1,∞), it follows that

$$D_{q}^{X}(s∥t)=\frac{1}{q-1}\log\sum_{i=1}^{n}s{\left(x_{i}\right)}^{q}t{\left(x_{i}\right)}^{1-q}\geq 0.$$

Let q∈(0,1). Then the function φ is concave, and therefore we get $\sum_{i=1}^{n}s{\left(x_{i}\right)}^{q}t{\left(x_{i}\right)}^{1-q}\leq 1,$ and consequently

$$\log\sum_{i=1}^{n}s{\left(x_{i}\right)}^{q}t{\left(x_{i}\right)}^{1-q}\leq \log1=0.$$

Since $\frac{1}{q-1}<0,$ for q∈(0,1), it follows that

$$D_{q}^{X}(s∥t)=\frac{1}{q-1}\log\sum_{i=1}^{n}s{\left(x_{i}\right)}^{q}t{\left(x_{i}\right)}^{1-q}\geq 0.$$

The equality in (14) holds if and only if $\frac{t(x_{i})}{s(x_{i})}$ is constant, for i=1,2,…,n, i.e., if and only if $t(x_{i})=k\cdot s(x_{i}),$ for i=1,2,…,n. By summing over all i=1,2,…,n, we get $\sum_{i=1}^{n}t(x_{i})=k\cdot \sum_{i=1}^{n}s(x_{i}),$ which implies that k=1. Hence $s(x_{i})=t(x_{i}),$ for i=1,2,…,n. Therefore, we conclude that $D_{q}^{X}(s∥t)=0$ if and only if $s(x_{i})=t(x_{i}),$ for i=1,2,…,n. ☐

**Corollary** **3.** *Let* s *be a state on a product MV-algebra* (A,⋅), *and* $X=(x_{1},x_{2},\ldots ,x_{n})$ *be a partition in* (A,⋅) *such that* $s(x_{i})>0,$ *for* i=1,2,…,n. *Then* $H_{q}^{s}(X)\leq \log n$ *with the equality if and only if the state s is uniform over X.*

**Proof.**  Let t:A→[0,1] be a state uniform over *X, i.e.,* $t(x_{i})=\frac{1}{n},$ for i=1,2,…,n. Then according to Theorems 14 and 13, it holds

$$0\leq D_{q}^{X}(s∥t)=\log n-H_{q}^{s}(X),$$

which implies that $H_{q}^{s}(X)\leq \log n.$ Since the equality $D_{q}^{X}(s∥t)=0$ applies if and only if $s(x_{i})=t(x_{i}),$ for i=1,2,…,n, the equality $H_{q}^{s}(X)=\log n$ holds if and only if the state *s* is uniform over *X*. ☐

**Example** **8.** *Let us consider any product MV-algebra*(A,⋅),*and states*$s_{1},s_{2},s_{3}$*defined on it. Let*x∈A*with*$s_{i}(x)=p_{i},$*where*$p_{i}\in (0,1),$*for*i=1,2,3. *Then*$s_{i}(u-x)=1-p_{i},$*for*i=1,2,3. *Further, we consider the partition*X=(x,u−x)*in*(A,⋅).*Putting*$p_{1}=\frac{1}{2},$$p_{2}=\frac{1}{3},$$p_{3}=\frac{1}{4},$*and*q=2,*we obtain*

$$D_{2}^{X}(s_{1}∥s_{2})=\log\left[{\left(\frac{1}{2}\right)}^{2}\cdot {\left(\frac{1}{3}\right)}^{-1}+{\left(\frac{1}{2}\right)}^{2}\cdot {\left(\frac{2}{3}\right)}^{-1}\right]\dot{=}0.1699\;bit;$$

$$D_{2}^{X}(s_{1}∥s_{3})=\log\left[{\left(\frac{1}{2}\right)}^{2}\cdot {\left(\frac{1}{4}\right)}^{-1}+{\left(\frac{1}{2}\right)}^{2}\cdot {\left(\frac{3}{4}\right)}^{-1}\right]\dot{=}0.4150\;bit;$$

$$D_{2}^{X}(s_{2}∥s_{3})=\log\left[{\left(\frac{1}{3}\right)}^{2}\cdot {\left(\frac{1}{4}\right)}^{-1}+{\left(\frac{2}{3}\right)}^{2}\cdot {\left(\frac{3}{4}\right)}^{-1}\right]\dot{=}0.0525\;bit.$$

*For*$q=\frac{1}{2},$*we get*$D_{q}^{X}(s_{1}∥s_{2})\dot{=}0.04186$*bit;*$D_{q}^{X}(s_{1}∥s_{3})\dot{=}0.1$*bit;*$D_{q}^{X}(s_{2}∥s_{3})\dot{=}0.0122$*bit. Evidently, in both cases mentioned above,*

$$D_{q}^{X}(s_{1}∥s_{3})>D_{q}^{X}(s_{1}∥s_{2})+D_{q}^{X}(s_{2}∥s_{3}).$$

*This means that the triangle inequality for the Rényi divergence generally does not apply. The result means that it is not a metric in a true sense.*

**Theorem** **15.** *Let* s,t *be states on a given product MV-algebra* (A,⋅), *and* $X=(x_{1},x_{2},\ldots ,x_{n})$ *be a partition in* (A,⋅) *such that* $s(x_{i})>0,$ *and* $t(x_{i})>0,$ *for* i=1,2,…,n. *Then:*

*(i)*  0<q<1 *implies* $D_{q}^{X}(s∥t)\leq D_{X}(s∥t);$
*(ii)*  q>1 *implies* $D_{q}^{X}(s∥t)\geq D_{X}(s∥t),$

*where*

$$D_{X}(s∥t)=\sum_{i=1}^{n}s(x_{i})\cdot \log\frac{s(x_{i})}{t(x_{i})}.$$

**Proof.**  We prove the claims by applying the Jensen inequality to the concave function φ defined, for every x∈(0,∞), by φ(x)=logx. If we put $c_{i}=s(x_{i}),$ and $a_{i}={\left(\frac{s(x_{i})}{t(x_{i})}\right)}^{q-1},$ for i=1,2,…,n, in the inequality (6), we get

$$\begin{array}{c} \log\sum_{i=1}^{n}s{\left(x_{i}\right)}^{q}t{\left(x_{i}\right)}^{1-q}=\log\sum_{i=1}^{n}s(x_{i}){\left(\frac{s(x_{i})}{t(x_{i})}\right)}^{q-1}\\ \geq \sum_{i=1}^{n}s(x_{i})\log{\left(\frac{s(x_{i})}{t(x_{i})}\right)}^{q-1}=(q-1)\sum_{i=1}^{n}s(x_{i})\log\frac{s(x_{i})}{t(x_{i})}. \end{array}$$

(i) Suppose that 0<q<1. Then $\frac{1}{q-1}<0,$ and therefore we obtain
  $$D_{q}^{X}(s∥t)=\frac{1}{q-1}\log\sum_{i=1}^{n}s{\left(x_{i}\right)}^{q}t{\left(x_{i}\right)}^{1-q}\leq \sum_{i=1}^{n}s(x_{i})\log\frac{s(x_{i})}{t(x_{i})}=D_{X}(s∥t).$$
(ii) Suppose that q>1. Then $\frac{1}{q-1}>0,$ and we get
  $$D_{q}^{X}(s∥t)=\frac{1}{q-1}\log\sum_{i=1}^{n}s{\left(x_{i}\right)}^{q}t{\left(x_{i}\right)}^{1-q}\geq \sum_{i=1}^{n}s(x_{i})\log\frac{s(x_{i})}{t(x_{i})}=D_{X}(s∥t).$$

☐

To illustrate the result of previous theorem, let us consider the following example which is a continuation to Example 6.

**Example** **9.** *Consider the product MV-algebra* (A,⋅) *from Example 6 and the real functions* $F_{1},F_{2}$ *defined by* $F_{1}(x)=x,F_{2}(x)=x^{2},$ *for every* x∈[0,1]. *On the product MV-algebra* (A,⋅) *we define two states* $s_{1},s_{2}$ *by the formulas* $s_{i}(f)=\int_{0}^{1}f(x)dF_{i}(x),$ i=1,2, *for any element* f *of* A. *In addition, we consider the partition* $X=\left(I_{\left[0,\frac{1}{3}\right)},I_{\left[\frac{1}{3},1\right]}\right)$ *in* (A,⋅). *It can be easily calculated that it has the* $s_{1}$ *-state values* $\frac{1}{3},\frac{2}{3}$ *of the corresponding elements, and the* $s_{2}$ *-state values* $\frac{1}{9},\frac{8}{9}$ *of the corresponding elements. By using Formula (5), it can be calculated the Kullback–Leibler divergences* $D_{X}(s_{1}∥s_{2})\dot{=}0.25163$ *bit, and* $D_{X}(s_{2}∥s_{1})\dot{=}0.19281$ *bit. Further, by simple calculations we obtain:* $D_{2}^{X}(s_{1}∥s_{2})\dot{=}0.58496$ *bit,* $D_{2}^{X}(s_{2}∥s_{1})\dot{=}0.28951$ *bit,* $D_{1/3}^{X}(s_{1}∥s_{2})\dot{=}0.0707$ *bit, and* $D_{1/3}^{X}(s_{2}∥s_{1})\dot{=}0.07736$ *bit. As can be seen, for* $q=\frac{1}{3},$ *we have* $D_{q}^{X}(s_{1}∥s_{2})<$ $D_{X}(s_{1}∥s_{2}),$ $D_{q}^{X}(s_{2}∥s_{1})<D_{X}(s_{2}∥s_{1}),$ *and for* q=2, *we have* $D_{q}^{X}(s_{1}∥s_{2})>$ $D_{X}(s_{1}∥s_{2}),$ $D_{q}^{X}(s_{2}∥s_{1})>$ $D_{X}(s_{2}∥s_{1}).$ *The obtained results correspond to the claim of Theorem 15. Based on previous results, we can also see that the Rényi divergence* $D_{q}^{X}(s∥t),$ *as well as the Kullback–Leibler divergence* $D_{X}(s∥t),$ *is not symmetrical.*

## 6. Conclusions

The aim of this paper was to generalize the results concerning the Shannon entropy and Kullback-Leibler divergence in a product MV-algebra given in [22] and [24] to the case of Rényi entropy and Rényi divergence. The results are contained in Section 3, Section 4 and Section 5. In Section 3, we have introduced the concept of Rényi entropy $H_{q}^{s}(X)$ of a partition *X* in a product MV-algebra (A,⋅), and we examined properties of this entropy measure. In Section 4, we have defined the conditional Rényi entropy of partitions in the studied algebraic structure. It was shown that the proposed concepts are consistent, in the case of the limit of q→1, with the Shannon entropy of partitions defined and studied in [22]. Moreover, it was shown that the Rényi entropy $H_{q}^{s}(X)$ as well as the conditional Rényi entropy $H_{q}^{s}(X/Y)$ are monotonically decreasing functions of parameter q. In the final part of Section 4, we have defined the Rényi information about a partition *X* in a partition *Y* as an example for the further usage of the proposed concept of the conditional Rényi entropy. Section 5 was devoted to the study of Rényi divergence in (A,⋅). We have proved that the Kullback–Leibler divergence of states defined on a product MV-algebra can be derived from their Rényi divergence as the limiting case for q going to 1. Theorem 14 allows interpreting the Rényi divergence as a distance measure between two states (over the same partition) defined on a given product MV-algebra. In addition, we investigated the relationship between the Rényi entropy and the Rényi divergence (Theorem 13), as well as the relationship between the Rényi divergence and Kullback–Leibler divergence (Theorem 15), in a product MV-algebra.

In the proofs we used L’Hôpital’s rule, the triangle inequality of q-norm, and the Jensen inequality. To illustrate the results, we have provided several numerical examples.

As has been shown in Example 1, the model studied in this article generalizes the classical case; that is, the Rényi entropy and the Rényi divergence defined in this paper are a generalization of the classical concepts of Rényi entropy and Rényi divergence. On the other hand, MV-algebras enable to study more general situations. We note that MV-algebras can be for example interpreted by means of Ulam some games (see e.g., [35,36,37]). The obtained results could therefore be useful for the researches on this subject.

In Example 2, we have mentioned that the full tribe of fuzzy sets represents a special case of product MV-algebras; therefore, the results of the article can be immediately applied to this important class of fuzzy sets. We recall that by a fuzzy subset of a non-empty set Ω (cf. [38]), we understand any map f:Ω→[0,1]. The value f(ω) is interpreted as the degree of belongingness of ω∈Ω to the considered fuzzy set f. In [39], Atanassov has generalized the fuzzy sets by introducing the idea of an intuitionistic fuzzy set, a set having with each member a degree of belongingness as well as a degree of non-belongingness. From the application point of view, it is interesting that to a given class F of intuitionistic fuzzy sets can be constructed an MV-algebra A such that F can be embedded to A. Also, an operation of product on F can be introduced by such a way that the corresponding MV-algebra is a product MV-algebra. Therefore, all the results of this article can also be applied to the intuitionistic fuzzy case.
